# Rapid Quantification of Ceftobiprole in Human Plasma and Cerebrospinal Fluid by LC-MS/MS and Its Application in Patients with Central Nervous System Infections

**DOI:** 10.3390/molecules31081252

**Published:** 2026-04-10

**Authors:** Sabahat Ablimit, Wanzhen Li, Mengting Chen, Jing Zhang, Nanyang Li, Yaxin Fan, Muyassar Yasen, Mubarak Iminjan, Beining Guo

**Affiliations:** 1 College of Pharmacy, Xinjiang Medical University, Urumqi 830017, China; 17690925653@163.com (S.A.); 17590823792@163.com (M.Y.); 2 Institute of Antibiotics, Key Laboratory of Clinical Pharmacology of Antibiotics, National Clinical Research Center for Aging and Medicine, Professional Technical Service Platform for Comprehensive Preclinical Pharmacodynamic Evaluation and Clinical Translation of Antibacterial Agents, Joint Laboratory of Hospital & Enterprise for Pathogen Diagnosis of Drug-Resistant Bacterial lnfections and lnnovative Drug R&D, Huashan Hospital, Fudan University, Shanghai 200040, China; wanzhen_li@fudan.edu.cn (W.L.); chenmengting980818@163.com (M.C.); zhangj61@fudan.edu.cn (J.Z.); fanyaxin20080908@126.com (Y.F.); 3 Clinical Pharmacology Research Center, Huashan Hospital, Fudan University, Shanghai 200040, China; linanyang1213@163.com

**Keywords:** ceftobiprole, cerebrospinal fluid, liquid chromatography–tandem mass spectrometry, penetration, plasma

## Abstract

Ceftobiprole is a fifth-generation beta-cephalosporin with high inter-individual pharmacokinetic variability in critically ill patients. However, data on its pharmacokinetics and central nervous system (CNS) penetration are limited. This study developed and validated a rapid LC-MS/MS method for quantifying ceftobiprole in human plasma and CSF. Sample preparation involved protein precipitation of 50 µL aliquots. Analysis used gradient elution on an ACQUITY UPLC^®^ HSS T3 column (2.1 × 100 mm, 1.8 µm) with 0.2% formic acid and acetonitrile and was detected by positive ion electrospray, achieving a 3.5 min run time. The method was linear from 0.100 to 25.0 mg/L in plasma and 0.0500 to 15.0 mg/L in CSF. Intra- and inter-run precision and accuracy were within ±15% at all quality control levels. All validation parameters, including selectivity, matrix effects, recovery, and stability under various conditions, met acceptance criteria. Potential interference from the prodrug ceftobiprole medocaril was evaluated and found to be negligible. The method was successfully applied to samples from three patients, revealing a CSF penetration range of 11.9% to 36.5%. This validated LC-MS/MS method enables simple and rapid quantification of ceftobiprole in plasma and cerebrospinal fluid, filling the gap in data on its CNS penetration and supporting routine drug concentration monitoring in critically ill patients.

## 1. Introduction

Ceftobiprole is a fifth-generation cephalosporin with broad-spectrum activity against Gram-positive and Gram-negative bacteria, including methicillin-resistant *Staphylococcus aureus* (MRSA) [1,2,3]. Ceftobiprole medocaril sodium (Zevtera) is approved for multiple severe infections, including hospital-acquired and community-acquired pneumonia, acute bacterial skin and skin structure infections, and adult *Staphylococcus aureus* bloodstream infections [4]. In critically ill patients, the high pharmacokinetic (PK) variability of β-lactam antibiotics often necessitates routine therapeutic drug monitoring (TDM). This is particularly relevant for hospital-acquired central nervous system (CNS) infections, which frequently complicate neurosurgical procedures and carry attributable mortality rates of 4.4% to 33.3% [5]. Despite the need for effective antimicrobial therapies with reliable CNS penetration in this population, data on the pharmacokinetics and pharmacodynamics (PK/PD) of ceftobiprole in CNS infections remain scarce.

Ceftobiprole is administered as a prodrug, ceftobiprole medocaril, which is rapidly converted to the active drug in plasma with a remarkably low protein binding of approximately 16% [6]. As a time-dependent β-lactam antibiotic, its bactericidal efficacy is driven by the percentage of the dosing interval during which the free drug concentration remains above the minimum inhibitory concentration (%*f*T > MIC). While historical preclinical models have suggested conservative targets of 40–60% *f*T > MIC, recent clinical evidence strongly advocates for more aggressive thresholds (e.g., 100% *f*T > MIC or higher) in critically ill patients to ensure adequate tissue penetration and optimize outcomes [7]. Achieving such rigorous targets in deep compartments like the CNS further underscores the necessity of a TDM-guided approach for CNS infections.

Although approved for various serious infections, the clinical application of ceftobiprole in CNS infections remains poorly defined, largely due to limited evidence regarding its penetration across the blood–brain barrier. In a rabbit model of meningeal inflammation, the CSF penetration rate of ceftobiprole was reported to be approximately 16% [8]. A subsequent human case report measured a CSF concentration of 1.2 mg/L against a mean total plasma concentration of 9.74 ± 4.46 mg/L, yielding an estimated CSF-to-plasma ratio of 12% [9]. These preliminary data underscore the necessity for a validated analytical method to accurately measure ceftobiprole in human CSF and characterize its clinical pharmacokinetics. To date, the single reported case of ceftobiprole treatment for a CNS infection relied on an HPLC-UV method with a non-isotopic internal standard following solid-phase extraction, requiring 0.5–1 mL of sample [9].

Recent mass spectrometry-based assays have improved analytical efficiency. For example, an automated HPLC-MS/MS method was developed for ten plasma antibiotics [10], and an LC-MS/MS method was validated for ceftobiprole in the plasma and urine of older patients [11]. Another UPLC-MS/MS method simultaneously quantified cefiderocol and ceftobiprole in human plasma [12]. While these methods provide acceptable validation for TDM, their application in evaluating CNS penetration is limited, as they are restricted to plasma or urine and lack validation in CSF. Furthermore, their analytical throughput requires optimization for routine clinical use. More importantly, these existing assays overlook a critical pre-analytical challenge: ceftobiprole is administered as a prodrug (ceftobiprole medocaril). While the degradation kinetics of ceftobiprole have been studied using LC-MS/MS, the potential interference from the rapid ex vivo hydrolysis of the prodrug on the quantification of the active compound has not been systematically evaluated [12].

The aim of this study was to develop and validate a rapid and simple liquid chromatography–tandem mass spectrometry (LC-MS/MS) method for the quantification of ceftobiprole in human plasma and CSF. The method was subsequently applied to a clinical pharmacokinetic study in patients with central nervous system infections, providing a valuable tool to support pharmacokinetic studies and TDM in this population.

## 2. Results

### 2.1. Method Validation

#### 2.1.1. Selectivity and Specificity

**Figure 1:**
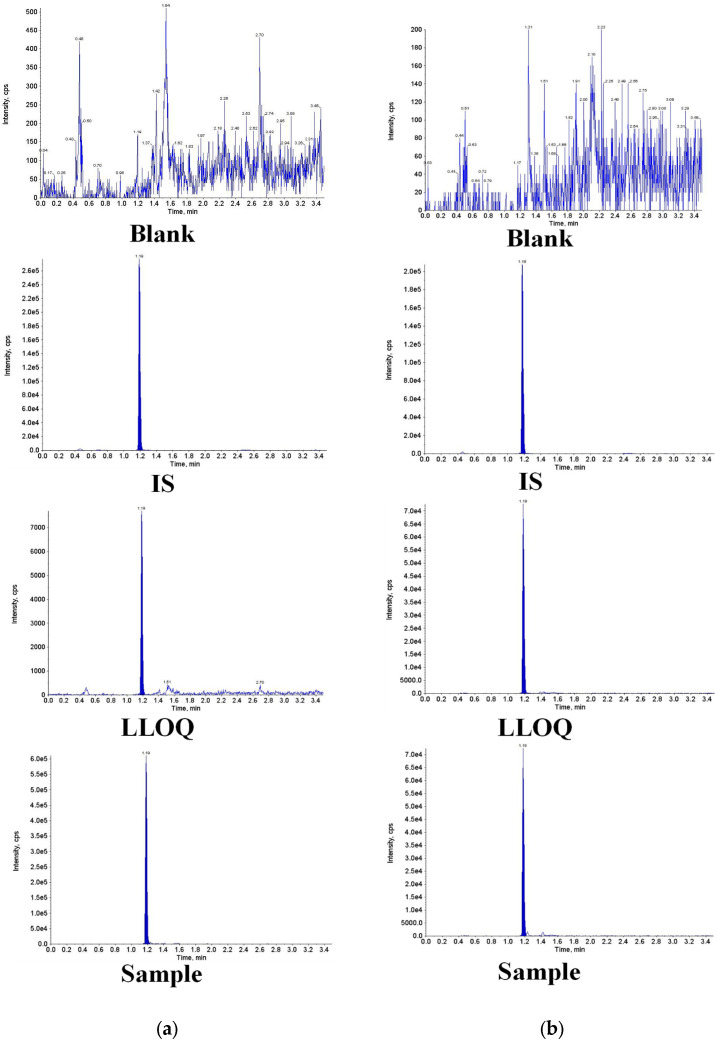
Representative chromatograms of ceftobiprole and internal standards (D4-ceftobiprole) acquired from (**a**) human plasma and (**b**) cerebrospinal fluid. LLOQ, lower limit of quantitation.

Chromatograms of blank, LLOQ, and pharmacokinetic samples demonstrated co-elution of the analyte and the IS, without interference from endogenous matrix components. Representative chromatograms of human plasma and CSF are shown in Figure 1. The chemical structures of ceftobiprole and its internal standard are shown in Figure S1.

#### 2.1.2. Linearity, Accuracy, and Precision

The calibration curves exhibited excellent linearity within the tested ranges: 0.100–25.0 mg/L for plasma and 0.0500–15.0 mg/L for CSF, with correlation coefficients (R^2^) exceeding 0.9900. The representative regression equations were as follows: plasma, y = 0.1588x − 0.0004 (R^2^ = 0.9995); CSF, y = 0.1582x + 0.0002 (R^2^ = 0.9984). For plasma samples, intra- and inter-batch accuracy ranged from 99.2% to 101.9%, with precision (CV) between 0.5% and 3.8%. For CSF samples, intra- and inter-batch accuracy ranged from 97.4% to 102.0%, with precision between 0.9% and 5.6% (Table 1).

According to the ICH M10 guideline, the LLOQ should be sufficiently sensitive to quantify drug concentrations at clinically relevant low levels. Based on reported pharmacokinetic data, plasma C_max_ is approximately 32.0 mg/L [13], and the estimated CSF C_max_ is ~3.84 mg/L, assuming a penetration ratio of 12% [9]. To ensure reliable measurement, LLOQ should at least cover 1/20 of C_max_, corresponding to 1.6 mg/L in plasma and 0.192 mg/L in CSF. In this study, the LLOQ was set at 0.1000 mg/L for plasma and 0.0500 mg/L for CSF, both of which meet these requirements.

#### 2.1.3. Matrix Effects and Extraction Recovery

Matrix effects and extraction recovery were evaluated in accordance with the ICH M10 guideline.

Matrix effects were assessed by analyzing low- and high-QC samples prepared from at least six different sources of blank matrices, including normal, hemolyzed, and lipemic plasma. Each matrix was evaluated in triplicate. The accuracy and precision for each matrix lot were within ±15% and ≤15%, respectively, indicating no significant matrix effect. Matrix effects of ceftobiprole in plasma (including hemolyzed and lipemic matrices) ranged from 100.5% to 104.7% at low and high concentrations and in CSF ranged from 96.9% to 105.6%.

Extraction recovery was determined by comparing the mean peak area of analytes in pre-spiked QC samples with that of post-extraction spiked samples at equivalent concentrations. Specifically, six replicates of QC samples at low, medium, and high concentrations were processed and compared with blank matrix extracts spiked after extraction. Extraction recoveries at low, medium, and high concentrations were 81.1–90.4% in plasma and 101.9–105.0% in CSF. The recovery of the IS was 85.9% in plasma and 102% in CSF. Detailed results are summarized in Table 2.

#### 2.1.4. Stability

To ensure that samples do not degrade during routine clinical handling, storage, and instrumental analysis, comprehensive stability conditions were evaluated. Stability testing demonstrated that ceftobiprole remained stable for at least 6 h in plasma and 16 h in CSF at room temperature, 15 days at −20 °C, over one year at −70 °C, through three freeze–thaw cycles at −70 °C, and for at least 48 h in the autosampler (4 °C) after sample preparation. The short- and long-term stability results in plasma and CSF are summarized in Table 3. In whole blood, ceftobiprole showed a recovery rate of approximately 97.3% after storage at room temperature for 2 h.

#### 2.1.5. Transformation of Prodrugs

During the infusion phase, transient levels of the prodrug (ceftobiprole medocaril) coexist with high concentrations of the active drug. To simulate this clinical scenario and evaluate the potential impact of ex vivo conversion on assay reliability, a fixed concentration of ceftobiprole medocaril (4.00 µg/mL) was added to the high-concentration QC. The accuracy of ceftobiprole quantified in plasma under different conditions was then evaluated to verify whether it remained within the acceptance range of 85–115%, indicating that the prodrug does not interfere with the assay (Table 4).

#### 2.1.6. Dilution Integrity

In actual clinical practice, patient samples frequently exhibit drug levels exceeding the assay’s upper limit of quantification (ULOQ). This is particularly common for samples drawn at peak concentration (C_max_) or from critically ill patients with impaired drug clearance. To ensure these high-concentration samples can be accurately measured without matrix effects, dilution integrity was assessed. The accuracy and precision of ceftobiprole in plasma and CSF after 10-fold dilution were 104.8% and 103.4%, respectively, indicating that the method is suitable for quantifying samples with concentrations above the upper limit of quantification (ULOQ). Diluted plasma QC samples remained stable for 15 days at both −20 °C and −70 °C, and diluted CSF QC samples were stable for 6 days at −20 °C and for over one year at −70 °C (Table 5).

### 2.2. Clinical Application

**Figure 2:**
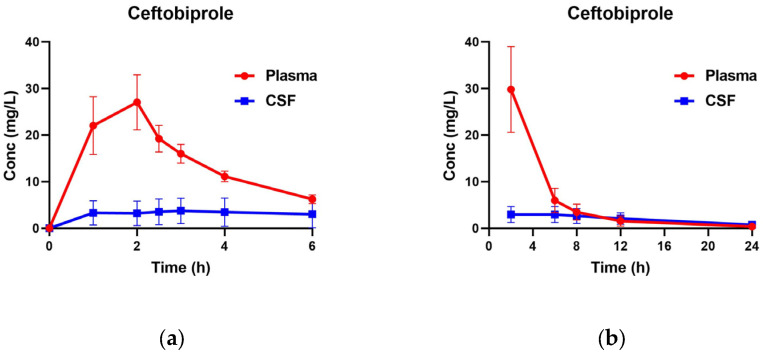
The plasma and CSF concentration–time curve of ceftobiprole in three patients with bacterial meningitis after intravenous infusion of 500 mg ceftobiprole medocaril sodium: (**a**) within 48 to 72 h after the first dose; (**b**) final administration. Conc, concentration.

The newly established and validated method was applied to the pharmacokinetic analysis of ceftobiprole in plasma and CSF of patients with suspected or confirmed meningitis following administration of ceftobiprole medocaril sodium. Representative chromatograms are provided in Figure 1. The concentration–time profiles of ceftobiprole in three patients after multiple doses of 500 mg every 6 h via 2-h infusion are presented in Figure 2. In plasma, the mean C_max_ was about 23.1 mg/L, reached 2 h post-infusion. In CSF, the mean C_max_ was 3.08 mg/L, with a slightly delayed peak at 3 h. Compared with plasma, CSF concentrations were generally more stable over time. Based on the CSF-to-plasma AUC_0–6h_ ratios, ceftobiprole CSF penetration ranged from 11.9% to 36.5% among the three patients. Non-compartmental PK analysis was performed using the Phoenix WinNonlin^®^ Version 8.1 software. The fraction unbound of ceftobiprole in plasma is 84% [6]. PK/PD targets were calculated based on the clinical breakpoint of *Staphylococcus aureus* for ceftobiprole (2 mg/L) [14]. Notably, 100% *f*T > MIC in plasma was achieved in all three patients, while values of 0%, 39.15%, and 100% *f*T > MIC were observed in CSF, indicating the high variability of CNS penetration.

## 3. Discussion

This study established a robust, rapid, and reproducible LC-MS/MS method for the quantitative determination of ceftobiprole in human plasma and CSF. The calibration range was 0.100–25.0 mg/L for plasma and 0.0500–15.0 mg/L for CSF. To place the validated ranges in a clinical context, reported pharmacokinetic data indicate that C_max_ is 32.0 mg/L following standard dosing [13], while CSF exposure is markedly lower (~3.84 mg/L) based on an estimated penetration ratio of 12% [9]. These observations show that the present method adequately covers clinically relevant concentrations in both matrices.

Previous LC–MS/MS methods for ceftobiprole quantification have predominantly focused on plasma analysis. For example, a UPLC–MS/MS method employed single-step protein precipitation with an analytical run time of approximately 5 min for quantifying ceftobiprole (in combination with cefiderocol) in human plasma [12]. Similarly, a recent LC–MS/MS method quantified ceftobiprole in plasma and urine using protein precipitation and isotope-labeled internal standards [11] but did not include CSF. In addition, earlier multi-analyte LC–MS/MS assays typically reported analytical run times of approximately 5–7 min per sample [10]. Although these methods demonstrate acceptable analytical performance, they are primarily limited to plasma (or plasma/urine), and validated methods for CSF remain scarce, restricting their application in evaluating CNS penetration. In comparison, the present study developed and validated LC–MS/MS methods for the quantification of ceftobiprole in both plasma and CSF using separate calibration and validation procedures for each matrix. The methods feature a shorter run time (3.5 min), simple protein precipitation with a small sample volume (50 µL), and suitable linear ranges (0.100–25.0 mg/L in plasma and 0.0500–15.0 mg/L in CSF). These characteristics make the approach more suitable for clinical pharmacokinetic studies, particularly for assessing CNS penetration in critically ill patients.

To optimize the analytical procedure, the water solubility of ceftobiprole was considered, which is relatively low (approximately 0.04 mg/mL, as determined experimentally). Solubility can be improved by adding an aqueous solubilizing agent such as dimethyl sulfoxide (DMSO) or dissolving the drug in an acidic medium. Because ceftobiprole exhibits greater stability under acidic conditions compared with neutral or alkaline solutions [15], and in reference to the 2025 edition of the CLSI M100™ guidelines [16], the stock solution was prepared in 1% DMSO, and the working solution was diluted with 1% formic acid in water. Regarding pre-analytical stability, in previous studies, 2 M citric acid was usually added during sampling to stabilize the prodrug [17]. However, this procedure increases the complexity of clinical operations. Ceftobiprole medocaril is rapidly hydrolyzed by nonspecific plasma lipases to its active form, ceftobiprole. As a result, the prodrug is generally undetectable in plasma except during infusion, when only minimal levels may transiently coexist with high concentrations of the active drug [18]. To simulate this condition during method validation, we incorporated a fixed concentration of 4.0 mg/L ceftobiprole medocaril into the high-concentration quality control samples. This design enabled us to evaluate whether the conversion of ceftobiprole medocaril to ceftobiprole in mixed samples would interfere with the accurate quantification of ceftobiprole, and the results confirmed that the assay remained robust. Having verified that prodrug conversion did not compromise ceftobiprole measurement, citric acid was omitted in subsequent clinical sampling to simplify procedures. Whole blood was directly collected in EDTA-K2 tubes, and CSF was collected using transparent sampling tubes. Furthermore, another study reported non-enzymatic hydrolysis of ceftobiprole, with the conversion rate shown to be temperature-dependent. In 0.9% sodium chloride solution, ceftobiprole remained stable for up to 24 h at both room temperature and under refrigeration, retaining over 95% of its initial concentration. In contrast, its stability in glucose solution was markedly reduced [13]. The mechanism of this non-enzymatic degradation and its clinical implications warrant further investigation.

Clinically, pneumonia, endocarditis, and bloodstream infections caused by MRSA are associated with high mortality. Recently, new agents such as ceftobiprole have become available for the treatment of MRSA infections. While trials like the ERADICATE randomized controlled trial demonstrated that ceftobiprole is an effective treatment option for MRSA bacteremia, including endocarditis [19,20,21,22], and no new safety concerns were identified in real-world use of ceftobiprole, including in patients with severe renal impairment, hepatic impairment, or immunocompromised status [23], individualizing therapy remains crucial. TDM of these agents may optimize efficacy and safety; however, their implementation remains challenging in many hospitals [24].

Despite these findings, data regarding ceftobiprole penetration into the CNS and its pharmacokinetics in patients with CNS infections remain limited. To date, only a single case has been reported in which ceftobiprole was used to treat a CNS infection. In that case, the regimen was 500 mg every 8 h with a prolonged 3 h infusion, and drug quantification was performed by HPLC-UV using a non-isotopic internal standard after solid-phase extraction, requiring 0.5–1 mL of sample [9]. In our study, the newly developed quantitative LC–MS/MS method was successfully applied to assess the pharmacokinetics of ceftobiprole in three patients with CNS infection. The mean plasma C_max_ was 23.1 mg/L, and the mean CSF C_max_ was 3.08 mg/L, with CSF penetration ranging from 11.9% to 36.5%. Given the complexity of CNS infections and the high interpatient variability in pharmacokinetics, sample sizes remain limited, and further studies with larger cohorts are warranted to better characterize the PK profile of ceftobiprole in this population.

## 4. Materials and Methods

### 4.1. Chemicals and Reagents

Ceftobiprole (purity 92.0%) and its prodrug ceftobiprole medocaril sodium (purity 90.3%) were provided by Shenyang Sanjiu Pharmaceutical Co., Ltd. (Shenyang, China). The internal standard (IS) D4-ceftobiprole (purity 96.87%) was supplied by TOREF. Methanol (HPLC grade), acetonitrile (HPLC grade), and formic acid (GR grade) were purchased from Merck (Darmstadt, Germany). Ultrapure water was generated by a Millipore^®^ ultrapure water system. Blank biological matrices, including whole blood and plasma, were collected from healthy volunteers, whereas a blank CSF matrix was obtained from patients who had not received ceftobiprole medocaril sodium.

### 4.2. LC-MS/MS Conditions

The LC-MS/MS system consisted of a Shimadzu LC-30A (Nakagyo-ku, Japan) coupled with an AB SCIEX Triple Quad 5500 mass spectrometer (Framingham, MA, USA). Chromatographic separation was achieved using an ACQUITY UPLC^®^ HSS T3 (Waters, Milford, MA, USA, 2.1 mm × 100 mm; 1.8 µm). The mobile phase was 0.2% formic acid acetonitrile (mobile phase A) and 0.2% formic acid water (mobile phase B), delivered at a flow rate of 0.5 mL/min. The gradient elution was performed as follows: 0–0.8 min, 95–50% B; 0.8–1.5 min, 50–10% B; 1.5–2.5 min, 10% B; 2.5–3.0 min, 10–95% B; 3.00–3.50 min, 95% B. The column temperature was set at 40.0 °C, and the injection volume was 3 µL.

For mass spectrometry detection, analytes were ionized using an electrospray ionization (ESI) source in positive mode and monitored in multiple reaction monitoring (MRM) mode. The ion-spray voltage and source temperature were set at 5500 V and 550 °C, respectively. Nebulizer gas (GS1), heater gas (GS2), and curtain gas were set at 55, 55, and 35 psi, respectively. The quantifier ion transitions were *m*/*z* 535.2 → 264.2 for ceftobiprole and *m*/*z* 539.2 → 268.0 for D4-ceftobiprole. The qualifier ion transition was *m*/*z* 535.2 → 307.9 for ceftobiprole and *m/z* 539.2 → 312.3 for D4-ceftobiprole. The declustering potential (DP) was 120 V, and the collision energy (CE) was 30 V for all analytes (Figure S2).

### 4.3. Preparation of Calibration Standard and Quality Control Samples

Ceftobiprole and IS were accurately weighed and dissolved in 1% formic acid in dimethyl sulfoxide to obtain stock solutions at 4000 mg/L and 1000 mg/L, respectively. The prodrug ceftobiprole medocaril sodium was dissolved in water to prepare 1000 mg/L stock solution.

For plasma analysis, the ceftobiprole stock solution was diluted with 1% formic acid in water to obtain working solutions at 2.00, 4.00, 20.0, 50.0, 100, 200, 300 and 500 mg/L for calibration and 6.00, 180, and 400 mg/L for quality control (QC). The IS stock solution was diluted to a working solution of 8.00 mg/L using the same solvent. Calibration and QC samples were prepared by spiking blank plasma at a ratio of 1:20 (*v*/*v*) with the corresponding working solutions, yielding final concentrations of 0.100–25.0 mg/L for calibration and 0.300, 9.00, and 20.0 mg/L for QC.

For CSF analysis, calibration working solutions were prepared at 1.00, 2.00, 4.00, 20.0, 50.0, 100, 200, and 300 mg/L and QC solutions at 3.00, 120, and 240 mg/L. The IS was also diluted to 8.00 mg/L. Calibration and QC samples were prepared by spiking blank CSF at a ratio of 1:20 (*v*/*v*) with the corresponding working solutions, yielding final concentrations of 0.0500–15.0 mg/L for calibration and 0.150, 6.00, and 12.0 mg/L for QC. All solutions were prepared based on defined dilution ratios, and volumes were adjusted proportionally as required.

### 4.4. Sample Preparation

Plasma and CSF samples were prepared by mixing 50 µL of matrix with 50 µL IS working solution, followed by protein precipitation using 300 µL of methanol/acetonitrile (1:1, *v*/*v*). After vortexing and centrifugation at 12,000 rpm for 10 min, 100 µL of the supernatant was diluted with 400 µL of water in a 96-well plate, and 3 µL was injected into the LC-MS/MS system for analysis.

### 4.5. Method Validation

Method validation was performed in accordance with the ICH guidelines for bioanalytical method validation (M10) and validation of analytical procedures Q2(R2) [25,26]. Calibration curves were constructed using least-squares linear regression of ceftobiprole/IS peak area ratios versus concentration, with a weighting factor of 1/x^2^, and were required to achieve a correlation coefficient (r) greater than 0.98. Intra- and inter-batch accuracy and precision were assessed at the LLOQ, low (QCL), medium (QCM), and high (QCH) quality control levels across three independent batches on different days.

Selectivity was evaluated to confirm the absence of interference from endogenous substances. Blank plasma samples were obtained from healthy volunteers and blank CSF samples from patients without exposure to ceftobiprole, representing six independent biological sources, and were analyzed for potential interferences at the retention times of ceftobiprole and the IS. For plasma, additional evaluations were performed in hemolyzed and lipemic matrices. Because ceftobiprole medocaril sodium is rapidly converted to ceftobiprole by nonspecific plasma lipases and is detectable only during infusion, potential interference from the prodrug was not considered. Instead, specificity was further evaluated by examining potential ion signal overlap between ceftobiprole and its internal standard. Matrix effects were evaluated at the QCL and QCH levels in plasma and CSF from six volunteers, including hemolyzed and lipemic plasma.

Extraction recovery was determined at QCL, QCM, and QCH levels. QC samples (n = 6) were prepared using a mixed blank matrix. In parallel, blank matrices were pretreated and subsequently spiked with drug solutions (n = 6), and recovery was calculated as the ratio of analyte peak areas between these two sets of samples. Stability was evaluated under different storage conditions, including room temperature, −20 °C, and −70 °C, and in the autosampler, after sample preparation. Stability was confirmed when concentrations remained within 85–115% of nominal values.

All samples used for method validation were prepared by spiking known concentrations of ceftobiprole and internal standard into blank plasma (from healthy volunteers) and blank CSF (from patients without prior exposure to ceftobiprole). No clinical samples from patients receiving ceftobiprole were used during method validation.

### 4.6. Transformation of the Prodrug Ceftobiprole Medocaril Sodium in the Blood

Considering that the prodrug is detectable only during infusion, stability testing was performed only in plasma samples at high concentrations. To evaluate whether conversion of the prodrug affects ceftobiprole quantification, ceftobiprole medocaril sodium was added to the QCH plasma sample at a fixed concentration of 4.00 mg/L. The resulting ceftobiprole concentration was measured to confirm that it remained within 85–115% of the nominal value. Based on this consideration, stability testing of the prodrug was performed only in high-concentration plasma samples.

### 4.7. Clinical Application

**Figure 3:**
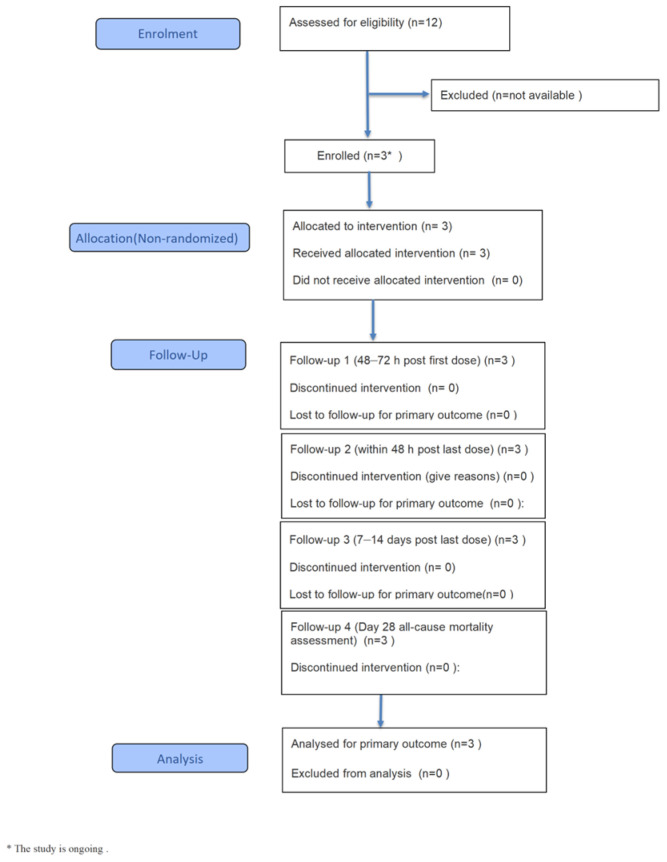
The flow diagram of the clinical study.

This study was a prospective, single-center, open-label, multi-dose intravenous administration study. Adult patients, regardless of gender, who were clinically diagnosed or suspected of having bacterial meningitis or ventriculitis after neurosurgery were enrolled for empirical or therapeutic use of ceftobiprole (Figure 3). A total of three patients were enrolled, and microorganisms isolated from all CSF samples were confirmed to be susceptible to ceftobiprole. Ceftobiprole medocaril sodium was administered by continuous intravenous infusion at 500 mg approximately every 6 h, with infusion lasting 2 h. The study protocol and informed consent form were reviewed and approved by the Ethics Committee of Huashan Hospital, Fudan University (No. 2024-783), and all participants provided written informed consent.

Serial blood samples were collected in EDTA-K2 tubes before the first dose (pre-dose) and within 48 to 72 h after the first dose. Six samples were collected during each dosing interval at 1, 2, 2.5, 3, 4, and 6 h post-dose. Additional samples were collected after the final administration at 2, 6, 8, 12, and 24 h post-dose. Blood samples (2 mL each) were centrifuged at 2000× *g* for 10 min at 4 °C within 1.5 h of collection. CSF samples (2 mL each) were collected at the same time points as plasma. Plasma and CSF samples were stored at −70 ± 10 °C until analysis.

## 5. Conclusions

This study developed a simple and rapid LC-MS/MS method for quantifying ceftobiprole in plasma and cerebrospinal fluid, which was successfully applied to clinical pharmacokinetic analysis. The method requires only 50 µL of sample without the need for stabilizers. Protein precipitation was employed for sample preparation, enabling fast and reliable quantification of ceftobiprole. The method was successfully applied to pharmacokinetic analysis in patients with central nervous system infections, allowing quantification of plasma and CSF concentrations and estimation of CSF penetration ranging from 11.9% to 36.5%. This approach may facilitate pharmacokinetic studies of ceftobiprole in patients with meningitis and other infectious diseases.

## Figures and Tables

**Table 1:** Accuracy and precision of ceftobiprole in human plasma and CSF.

Matrix	Nominal Conc (mg/L)		Intra-Batch (n = 5)			Inter-Batch (n = 16)		
			Mean Measured Conc (mg/L)	Accuracy (%)	CV (%)	Mean Measured Conc (mg/L)	Accuracy (%)	CV (%)
Plasma	LLOQ	0.100	0.102	101.8	2.1	0.102	101.9	3.8
	QCL	0.300	0.303	100.9	2.6	0.300	100.1	2.7
	QCM	9.00	8.98	99.8	1.3	8.93	99.2	1.9
	QCH	20.0	20.1	100.5	0.5	19.9	99.5	1.7
CSF	LLOQ	0.0500	0.0494	98.8	6.7	0.0487	97.4	5.6
	QCL	0.150	0.152	101.3	3.7	0.152	101.3	4.5
	QCM	6.00	6.04	100.7	0.9	6.12	102.0	2.1
	QCH	12.0	12.0	100.0	2.5	12.1	100.8	2.1

Conc, concentration; CV, coefficient of variation; LLOQ, lower limit of quantitation; QCL, low quality control; QCM, medium quality control; QCH, high quality control; CSF, cerebrospinal fluid.

**Table 2:** Matrix effect for the determination of ceftobiprole in human plasma and CSF.

Matrix	Samples	Matrix Effect (%)	Extraction Recovery (%)	
			Mean Recovery (%)	CV (%)
Plasma	QCL	100.5–102.3	90.4	3.1
	QCM	/	81.1	4.2
	QCH	100.6–104.7	84.3	2.7
	IS (D4-ceftobiprole)	/	85.9	5.9
CSF	QCL	97.6–105.6	105.0	3.4
	QCM	/	102.4	2.8
	QCH	96.9–102.8	101.9	4.7
	IS (D4-ceftobiprole)	/	102.0	4.0

CV, coefficient of variation; QCL, low quality control; QCM, medium quality control; QCH, high quality control; IS, internal standard; CSF, cerebrospinal fluid.

**Table 3:** Stability in human plasma and CSF at different conditions.

Matrix	Conditions	QCL		QCH	
		Accuracy (%)	CV (%)	Accuracy (%)	CV (%)
Plasma	Room temperature 6 h	96.0	2.0	92.1	1.9
	Post-preparation (autosampler, 4 °C) 48 h	95.7	3.2	99.7	3.9
	Three freeze–thaw cycles (−70 °C)	90.5	6.6	98.2	3.5
	Long-term 15 days (−20 °C)	96.7	3.3	97.7	2.6
	Long-term 386 days (−70 °C)	85.7	2.2	86.0	1.2
CSF	Room temperature 16 h	94.4	1.8	92.8	0.5
	Post-preparation (autosampler, 4 °C) 48 h	103.3	4.2	99.0	1.4
	Three freeze–thaw cycles (−70 °C)	100.9	0.4	101.4	1.3
	Long-term 15 days (−20 °C)	88.7	2.0	93.6	1.9
	Long-term 378 days (−70 °C)	95.3	1.4	96.9	2.0

CV, coefficient of variation; QCL, low quality control; QCH, high quality control; CSF, cerebrospinal fluid.

**Table 4:** The stability of ceftobiprole in the high-concentration samples of ceftobiprole mixed with ceftobiprole medocaril sodium.

Matrix	Conditions	QCH	
		Accuracy (%)	CV (%)
Plasma	Room temperature 6 h	111.7	0.3
	Post-preparation (autosampler, 4 °C) 50 h	102.0	2.0
	Three freeze–thaw cycles (−70 °C)	106.5	0.6
	Long-term 15 days (−20 °C)	107.7	1.2
	Long-term 386 days (−70 °C)	96.2	0.3

CV, coefficient of variation; QCH, high quality control.

**Table 5:** Dilution of ceftobiprole in human plasma and CSF.

Matrix	Nominal Conc (mg/L)	Conditions	Accuracy (%)	CV (%)
Plasma	80.0	/	104.8	1.7
		Long-term 15 days (−20 °C)	94.4	2.2
		Long-term 386 days (−70 °C)	88.9	1.5
CSF	50.0	/	103.4	1.9
		Long-term 6 days (−20 °C)	85.5	2.1
		Long-term 378 days (−70 °C)	100.2	1.1

Conc, concentration; CV, coefficient of variation.

## Data Availability

The data supporting this study’s findings are available from the corresponding author, B.G., upon reasonable request.

## Supplementary Materials

The following supporting information can be downloaded at: https://www.mdpi.com/article/10.3390/molecules31081252/s1. Figure S1: The chemical structures of ceftobiprole and its internal standard (D4-ceftobiprole); Figure S2: MS/MS product ion spectra of ceftobiprole and its internal standard (D4-ceftobiprole).

## Abbreviations

The following abbreviations are used in this manuscript:

ABSSSI	Acute bacterial skin and skin structure infection
CNS	Central nervous system
CSF	Cerebrospinal fluid
DMSO	Dimethyl sulfoxide
ESI	Electrospray ionization
HAP	Hospital-acquired pneumonia
IS	Internal standard
LLOQ	Lower limit of quantification
MRM	Multiple reaction monitoring
MRSA	Methicillin-resistant *Staphylococcus aureus*
MS-CoNS	Methicillin-sensitive coagulase-negative *staphylococci*
MSSA	Methicillin-sensitive *Staphylococcus aureus*
QC	Quality control
TDM	therapeutic drug monitoring
UPLC-MS/MS	Ultra-performance liquid chromatography–tandem mass spectrometry
