# Supplementary material for: Rapid Quantification of Ceftobiprole in Human Plasma and Cerebrospinal Fluid by LC-MS/MS and Its Application in Patients with Central Nervous System Infections
Source: Molecules. 2026 Apr 10;31(8):1252. doi: 10.3390/molecules31081252 (PMC13119386; doi:10.3390/molecules31081252)
Supplement: Supplementary file 1 [file molecules-31-01252-s001.zip › molecules-4176288-supplementary.pdf]

Supplementary materials

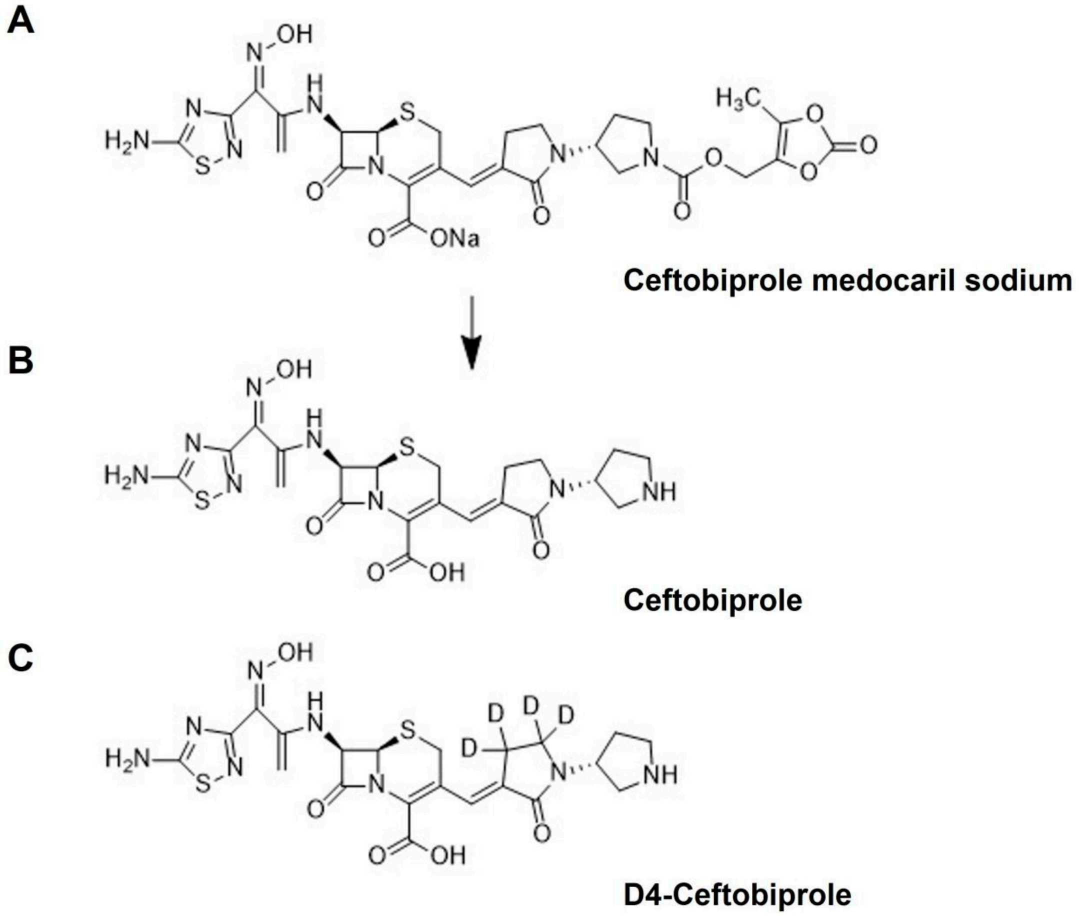

Figure S1. The chemical structures of ceftobiprole and its internal standard (D4-ceftobiprole). (A) Chemical structure of ceftobiprole medocaril sodium. (B) Chemical structure of ceftobiprole. (C) Chemical structure of the deuterated internal standard, D4-ceftobiprole.

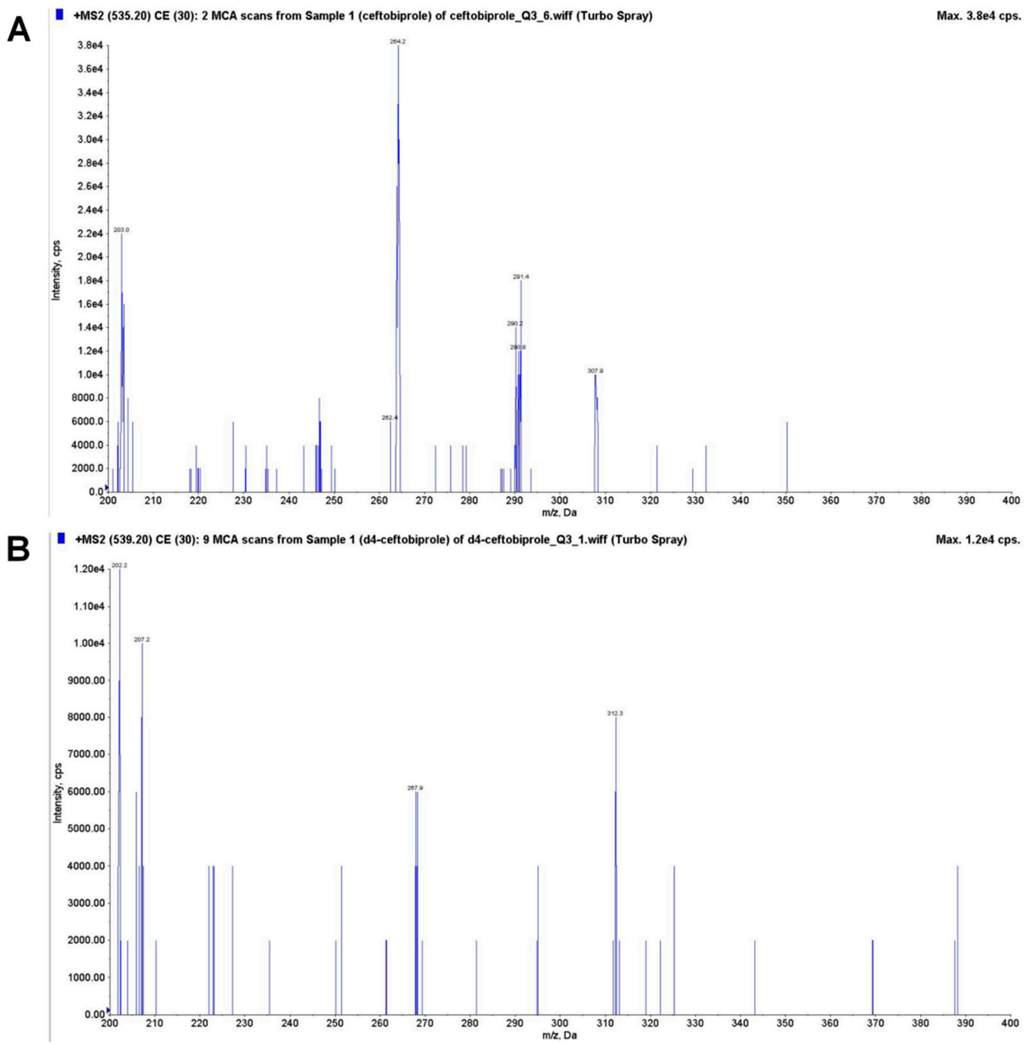

Figure S2. MS/MS product ion spectra of ceftobiprole and its internal standard (D4-ceftobiprole). (A) Ceftobiprole (*m*/*z* 535.2 → 264.2) (*m*/*z* 535.2 → 307.9); (B) D4-ceftobiprole (*m*/*z* 539.2 → 268.0) (*m*/*z* 539.2 → 312.3). Minor mass deviations for fragment ions are considered acceptable within the context of method validation and instrument accuracy.
